# Genome-wide analysis of the C2H2 zinc finger protein gene family and its response to salt stress in ginseng, *Panax ginseng* Meyer

**DOI:** 10.1038/s41598-022-14357-w

**Published:** 2022-06-17

**Authors:** Yue Jiang, Lingyu Liu, Zhaoxi Pan, Mingzhu Zhao, Lei Zhu, Yilai Han, Li Li, Yanfang Wang, Kangyu Wang, Sizhang Liu, Yi Wang, Meiping Zhang

**Affiliations:** 1 College of Life Science, Jilin Agricultural University, Changchun, 130118 Jilin China; 2 Jilin Engineering Research Center for Ginseng Genetic Resources Development and Utilization, Jilin Agricultural University, Changchun, 130118 Jilin China; 3 College of Chinese Medicinal Materials, Jilin Agricultural University, Changchun, 130118 Jilin China

**Keywords:** Genetics, Genomics, Transcriptomics

## Abstract

The C2H2 zinc finger protein (C2H2-ZFP) gene family plays important roles in response to environmental stresses and several other biological processes in plants. Ginseng is a precious medicinal herb cultivated in Asia and North America. However, little is known about the C2H2-ZFP gene family and its functions in ginseng. Here, we identified 115 C2H2-ZFP genes from ginseng, defined as the *PgZFP* gene family. It was clustered into five groups and featured with eight conserved motifs, with each gene containing one to six of them. The family genes are categorized into 17 gene ontology subcategories and have numerous regulatory elements responsive to a variety of biological process, suggesting their functional differentiation. The 115 *PgZFP* genes were spliced into 228 transcripts at seed setting stage and varied dramatically in expression across tissues, developmental stages, and genotypes, but they form a co-expression network, suggesting their functional correlation. Furthermore, four genes, *PgZFP31*, *PgZFP78-01*, *PgZFP38*, and *PgZFP39-01*, were identified from the gene family that were actively involved in plant response to salt stress. These results provide new knowledge on origin, differentiation, evolution, and function of the *PgZFP* gene family and new gene resources for C2H2-ZFP gene research and application in ginseng and other plant species.

## Introduction

Ginseng, as a valuable medicinal herb, has widely been used for human health food, medicine, and cosmetics. Its main bioactive ingredients, ginsenosides, have been shown to prevent, inhibit, and treat several types of cancers^1–3^. Therefore, ginseng genome research has been pursued extensively in the recent years. Xu et al.^4^ reported the first draft of the ginseng genome. Kim et al.^5^ released the draft of the ginseng genome for another genotype, from which 59,352 genes were identified. Recently, Wang et al.^6^ reported a chromosome-sized assembly of the ginseng genome for the third genotype, from which 65,913 genes were identified. Wang et al.^7^ sequenced the transcriptome of a 4-year-old ginseng plant expressed in 14 tissues, assembled 248,992 transcript unigenes spliced from 130,557 gene models and quantified their expressions in the 14 tissues. These genomic and transcriptomic resources have provided useful tools for genome-wide identification and characterization of genes important for ginseng research and breeding.

The C2H2 zinc finger protein (ZFP) gene family has been shown to play important roles in plant response to abiotic and biotic stresses, plant growth and development, and hormone signal transduction. For instance, the over-expression of an Arabidopsis C2H2-ZFP gene, *ZAT18*, improved drought tolerance^8^. The rice *bsr-d1* gene encoding a C2H2 transcription factor increased disease resistance and immunity against rice blast^9^. The tomato *SlZFP2* gene participated in the regulation of ABA biosynthesis during fruit development, thereby inhibiting fruit development through the transcriptional inhibition of flowering regulator^10^. It has been reported that the C2H2-ZFP gene family actively takes part in plant response to salt stress, which is significant for crop production^11^. The expression of a tomato C2H2-ZFP gene, *SlZF3*, enhanced plant salt tolerance^12^. The expression of *Zoysia japonica ZjZFN1* in Arabidopsis improved seed germination, plant adaptability to salt stress, and the percentage of green cotyledons under the salt stress condition^13^. The *AZFs* and *STZ* genes in Arabidopsis had transcriptional repression activities, thus responding to salt stress^14^. The *OsZFP213* gene in rice was shown to interact with *OsMAPK3*, thus enhancing salt tolerance^15^. In soybean and Arabidopsis, *GmZAT4* played an important role in PEG and NaCl stress tolerance and ABA response^16^.

Therefore, the genes of the C2H2-ZFP gene family have been genome-wide identified and characterized in serval plant species, including Arabidopsis that contains 176 C2H2-ZFP genes^17^, rice (189)^18^, poplar (109)^19^, *Medicago truncatula* (218)^20^, maize (211)^21^, and soybean (321)^22^. The C2H2-ZFP gene family is usually expressed as X2CX2–4CX12HX2–8H, where X represents any amino acid, and the number indicates the number of any amino acids^23^. It is characterized with its C2H2 zinc finger protein that forms coordination bonds with two pairs of cysteine and histidine residues and further forms a compact finger-like tetrahedral structure through β hairpins and α helices^24^. In addition, the gene family contains a few conserved domains, including DNA binding domain, transcription regulatory domain, and protein interaction domain^23^. The DNA binding domain is featured by a highly conserved QALGGH motif in which every amino acid is important for its DNA binding activity. Absence of the QALGGH motif in a C2H2-ZFP gene may reduce ABA sensitivity, thus stomatal size^25^, and influence inflorescence development^26^. Another unique feature of the gene family is length variation of the spacer between two adjacent zinc fingers of the C2H2 zinc finger protein, which may also affect DNA binding^23^. Moreover, some C2H2-ZFPs contain a highly conserved amino acid sequence (L/FDLNL/FxP), known the EAR motif, in carboxy terminal region^27^. The EAR motif is the smallest known transcription repression domain. When a single residue in the EAR motif changed, the transcription repression ability was greatly reduced or disappeared^28^. The three DLN amino acids of the hexa-peptide motif is indispensable for transcriptional inhibition and the presence of at least two DLN residues produced maximum transcriptional inhibition^29^.

Nevertheless, the C2H2-ZFP gene family has not been reported in ginseng. In the present study, we identified 228 C2H2-ZFP gene transcripts spliced from 115 C2H2-ZFP genes from Jilin ginseng, defined *PgZFP* genes, and characterized them in genetic diversity, evolution, expression, functional differentiation, and gene × gene interaction. Furthermore, we identified four *PgZFP* genes involved in ginseng response to salt stress, thus confirming the roles of the family in plant response to salt stress. Therefore, the findings of this study provide a comprehensive insight into the *PgZFP* gene family in ginseng, knowledge, and gene resources necessary for advanced research and breeding in ginseng and related species.

## Materials and methods

### Plant materials

Three sets of Jilin ginseng plant materials were used for this study (Supplemental Fig. S1A) These plant materials included 14 tissues of a 4-year-old cv. Damaya plant, roots of 5-, 12-, 18-, 25-year-old cv. Damaya plants, and 4-year-old plant roots of 42 cultivars (hereafter referred as to genotypes) coded from S1 to S42 (Supplemental Table S1). The seeds of these plant materials were all from our laboratory, which are available upon request. The 14 tissues of the 4-year-old cv. Damaya plant included fiber root, leg root, main root epiderm, main root cortex, arm root, rhizome, stem, leaf peduncle, leaflet pedicel, leaf blade, fruit peduncle, fruit pedicel, fruit, and seed^7^. The roots of 5-, 12-, 18-, 25-year-old plants all were collected from a cultivar, Damaya. The 42 genotypes were collected from the origin and diversity center of ginseng, Jilin Province, China where over 65% of the world ginseng was produced. These genotypes were representatives of the genetic diversity of Jilin ginseng.

### Databases

To facilitate ginseng functional genomics research, we previously developed several databases of functional genes for Jilin ginseng. These databases included the gene sequence and expression database in the above 14 tissues of the 4-year-old plant (Database A)^7^, the gene sequence and expression database of the four different year-old plant roots (Database B)^7^, and the gene sequence and expression database of the 4-year-old plant roots of 42 genotypes (Database C)^30^. These databases were used for this study. Database A consists of 248,992 unique transcripts, with 59,092–113,456 unique transcripts per tissue. Database B contains 54,444–65,412 unique transcripts per year-old plant root. Database C is made of 48,211–79,134 unique transcripts per genotype. Moreover, three ginseng genome assemblies from three genotypes^4–6^ were also used for this study.

### Identification of *PgZFP* gene transcripts

Database A was used to identify the C2H2-ZFP genes in ginseng. First, we retrieved the C2H2-ZFP Hidden Markov Model (HMM) (protein family: PF00096) from the Pfam database (Pfam 33.1; https://pfam.xfam.org/)^31^, used as queries to search ginseng Database A for C2H2-ZFP genes, and identified the putative C2H2-ZFP genes in ginseng. Then, the identified ginseng putative C2H2-ZFP genes were imported into iTAK (http://bioinfo.bti.cornell.edu/tool/itak) to further confirm the putative C2H2-ZFP genes by C2H2-ZFP conservative domain search^32^. The putative C2H2-ZFP genes that were confirmed to have the C2H2-ZFP domains were considered as ginseng C2H2-ZFP-encoding genes and defined as *PgZFP* genes (Supplemental Table S2). Finally, the ORF finder (https://www.ncbi.nlm.nih.gov/orffinder/) was used to identify the ORFs of the *PgZFP* genes. The relevant physiological and biochemical indicators of their putative protein sequences were predicted, including isoelectric point (PI), molecular weight (Da), grand average of hydropathicity, instability index, and aliphatic index, using Protparam (https://web.expasy.org/protparam/).

### Distribution of the *PgZFP* gene family in the ginseng genome and its gene number variation within the ginseng species and synteny with Arabidopsis

We aligned the *PgZFP* genes identified above to three ginseng genome assemblies^4–6^ using Blastn to determine their distribution in the ginseng genome^6^ and the variation of the *PgZFP* gene family size among genotypes. The criteria of identity ≥ 99%, cover length ≥ 200 bp, and e-value ≤ 1.0E−100 were used for the alignment. The *PgZFP* genes were aligned to the Arabidopsis genome at criteria of identity ≥ 75%, cover length ≥ 80 bp, and e-value ≤ 1.0E−10, given that ginseng is distantly related with Arabidopsis (Supplemental Fig. S1B)^33^. The distribution of the gene family in the ginseng genome and its synteny with the Arabidopsis genome were constructed using the R-package Circlize^34^. The genes aligned to each genome assembly were compared to identify the pan- and core-transcriptomes of the gene family among the three ginseng genotypes.

### Duplication, phylogeny, and evolution of the *PgZFP* gene family

To determine the origin, evolution, and phylogeny of the *PgZFP* gene family, we screened for the ORFs of the transcripts of all 115 *PgZFP* genes using the NCBI ORF finder (https://www.ncbi.nlm.nih.gov/orffinder/), calculated the Ka/Ks ratio of the *PgZFP* genes recently duplicated, constructed their phylogenetic tree, and predicted their conserved domains. The transcripts with complete CDS were used for Ka/Ks calculation using the KaKs_Calculator^35^. The time of the gene duplication and divergence was estimated by T = Ks/2λ × 10^−6^, where Ks is synonymous nucleotide substitution rate and λ is the number of substitutions per synonymous site per generation, for which λ = 6.5 × 10^−9^ was used^36–38^.

The transcript that has the longest amino acid sequence for a *PgZFP* gene was used for constructing the phylogenetic tree of the gene family. The phylogenetic tree was constructed using the maximum-likelihood (ML) method of MEGA X^39^, with 1000 bootstrap replications. Forty-five C2H2-ZFPs from Arabidopsis were downloaded from Plant Transcription Factor Database (http://plntfdb.bio.uni-potsdam.de/v3.0/) and used as the outgroup and evolutionary reference.

MEME^40^ was used to predict the conserved domains in the *PgZFP* gene transcripts. The putative protein sequences of the 26 *PgZFP* genes that have the complete conservative domains were used as representatives for the conserved domain prediction of the genes. Motif length was set to 10–50 amino acids and the maximum number of motifs was set to 6, 8, and 10, respectively, while other parameters were set as default.

### Functional divergence of the *PgZFP* genes

Studies has documented that the types of gene cis-regulatory elements, such as promoters, are related to the biological functionality of the genes, such as plant responses to hormones, abiotic and biotic stresses, and plant growth and development. We estimated the functional differentiation and divergence of the genes in the *PgZFP* gene family by gene ontology (GO) categorization and cis-regulatory element sequence analysis. The *PgZFP* gene transcripts were GO categorized using Blast2GO V5.0^41^. The enrichment of the number of *PgZFP* gene transcripts categorized into each subcategory was tested by Chi-square test using the categorization of the transcripts expressed in the 4-year-old ginseng plant as the control^7^. The 1500-bp upstream sequences of the *PgZFP* genes were analyzed, the cis-regulatory elements of the genes were identified, and the types of the cis-regulatory elements were classified using PlantCARE database^42^.

### Expression characterization and co-expression network of the *PgZFP* gene family and its hub genes

We characterized the *PgZFP* gene family by analysis of the expressions, heatmaps, and co-expression networks of its gene transcripts in 14 tissues of a 4-year-old plant, in 5-, 12-, 18-, and 25-year-old plant roots, and in the 4-year-old plant roots of 42 genotypes. The expressions of the *PgZFP* gene transcripts were extracted from Databases A, B, and C, respectively. The expression heatmaps of the genes were constructed using an R language package for heatmap construction and visualization. The co-expression networks of the genes were constructed using the BioLayout *Express*^3D^ Version 3.2 software^43^. Furthermore, we tested the tendency that the *PgZFP* gene transcripts formed a co-expression network by Student’s *t*-test using 176 transcripts randomly selected from the 228 *PgZFP* gene transcripts identified in this study with the same numbers of transcripts randomly selected from Database A as a control. The test was carried out at a series of cutoff values from *P* ≤ 5.0E−02 to 1.0E−08, with 20 replicates per cutoff value. In addition, we identified the hub genes of the *PgZFP* gene family network using the following criteria: network cutoff *P*-value ≤ 0.001, connectivity ≥ 30, and the percentage of hub genes in the total number of genes in the network ≤ 5%. The connectivity of a gene in a network indicates not only the degree of its co-expression correlation with other genes, but also the number of genes with which it interacts in the network.

### Response of ginseng to salt stress

To examine the potential function of the *PgZFP* gene family in response to salt stress, we stressed ginseng with the NaCl salt using its adventitious roots as experimental materials. The 1-cm adventitious roots were cultured and stressed on the B5 medium containing 0 mM, 20 mM, 40 mM, and 80 mM NaCl at 25^ oC^ for 30 days. The lengths of the roots were measured, quickly frozen in liquid nitrogen, and stored at -80℃ for gene expression analysis.

### Relative expressions of the *PgZFP* genes under salt stress

We first analyzed the *PgZFP* genes by aligning them to the salt-responsive genes identified in *A. thaliana*, *STZ* and *AZF*. The *PgZFP* genes that were best aligned to the *STZ* and *AZF* genes were then selected and subjected to comparative expression analysis using the ginseng adventitious roots stressed with and without salt by real-time quantitative PCR (qPCR) to test whether the genes of the *PgZFP* gene family respond to salt stress. The qPCR primers of the selected *PgZFP* genes were designed, based on their sequences (Supplemental Table S3). Total RNA was isolated from the adventitious roots stressed with the above different NaCl concentrations using the Trizol method. mRNA was purified from the total RNA, and first-strand cDNA was synthesized and used as qPCR templates. The *CYP* gene was used as the reference gene^44^. qPCR was conducted using the Applied Biosystems 7500 Real-Time System (ABI, USA) and the Ultra SYBR Mixture Kit (Low ROX) (ComWin, Beijing, China). The 2^−ΔΔCT^ method was employed to determine the relative expressions of the *PgZFP* genes.

### Research involving plants

Authors confirm that all methods were performed in accordance with the relevant guidelines and regulations. All plant materials used in this study were from the authors’ laboratory.

## Results

### Identification of the *PgZFP* gene transcripts and the biophysical and biochemical properties of their putative proteins

A total of 228 *PgZFP* gene transcripts were identified, with a sequence length from 202 to 5327 bp and an average length of 1419 bp (Supplemental Table S2). These transcripts were spliced from 115 *PgZFP* genes, with 1 to 20 transcripts per gene and an average of 2 transcripts per gene. The molecular weights of the putative proteins of the *PgZFP* gene transcripts varied from 3808 to 177,151 Daltons (Da), their isoelectric point (PIs) ranged from 4.55 to 12.13, the maximum grand average of their hydropathicities was 1.253, and the minimum grand average of their hydropathicities was − 1.512. Since only 16 of the *PgZFP* gene transcripts had a positive grand average of hydropathicity, we speculated that most genes of the *PgZFP* gene family code hydrophilic proteins. The instability indices of the *PgZFP* putative proteins were between 18.90 and 88.75, with most of them being greater than 40.00, suggesting that most *PgZFP* genes in the gene family code unstable proteins (Supplemental Table S4).

### Distribution of the *PgZFP* gene family in the ginseng genome and its gene number variation within the ginseng species and synteny with Arabidopsis

**Figure 1:**
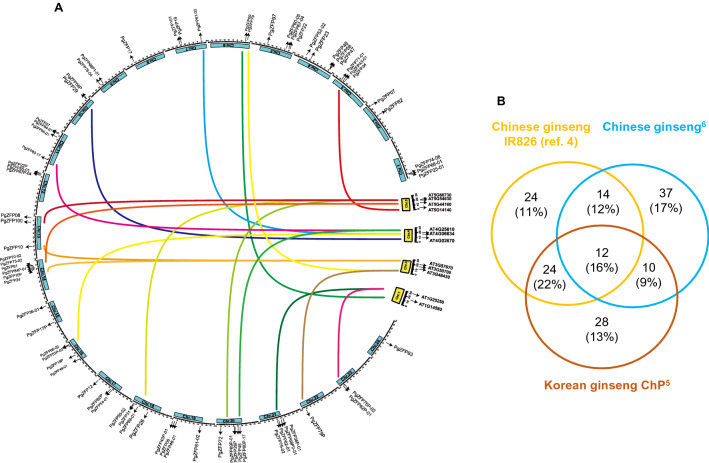
The distribution of the *PgZFP* gene family in the ginseng genome and its family size variation among ginseng genotypes. (**A**) The positions of the *PgZFP* gene family in the Chinese ginseng genome^6^ and its synteny with the Arabidopsis genome. The *PgZFP* genes were mapped to all 24 ginseng chromosomes indicated by light blue bars and their Arabidopsis orthologues were mapped to four of the five Arabidopsis chromosomes indicated by yellow bars. (**B**) Venn diagram showing the pan- and core-transcriptomes of the *PgZFP* gene family. The numbers in the Venn diagram indicate the numbers of the *PgZFP* genes in the family that are genotype-specific, shared between the genotypes, and common among all three genotypes. The percentage of the genes was calculated by the numbers of genes divided by the total number of the *PgZFP* genes (221) aligned to the genome of each genotype times 100.

Seventy-three of the 115 *PgZFP* genes identified in this study were mapped to all 24 chromosomes of the Chinese ginseng^6^, with each chromosome having 1–6 *PgZFP* genes. Seventeen of 73 *PgZFP* genes mapped to the Chinese ginseng genome^6^ were shown to be syntenic to 11 Arabidopsis orthologues in four of the five Arabidopsis chromosomes, Chromosomes 1, 3, 4, and 5, with each chromosome having 2–4 Arabidopsis orthologues (Fig. 1A). Of the 115 *PgZFP* genes identified in this study, 74 and 74 were aligned to the genomes of Chinese ginseng IR826 (Ref.^4^) and Korean ginseng ChP^5^, respectively. Comparative analysis showed that the pan-transcriptome of the gene family consisted of 149 *PgZFP* genes and the core-transcriptome of the gene family contained only 12 *PgZFP* genes, suggesting a wide variation of the gene family size among genotypes within *P. ginseng*.

### Gene duplication, phylogeny, and conserved motifs of the *PgZFP* gene family

We first estimated the duplication and selection mode of the *PgZFP* genes in the gene family. Analysis of 20 pairs of the *PgZFP* genes that have complete ORFs and have most recently duplicated revealed that these genes have been duplicated and diverged 18.0–40.7 million years ago (MYA). Approximately 55% of them have been subjected to purifying selection (Ka/Ks < 1.0 ± 0.1), 40% to neutral selection (Ka/Ks = 1.0 ± 0.1), and 5% to positive selection (Ka/Ks > 1.0 ± 0.1) (Supplemental Table S5).

**Figure 2:**
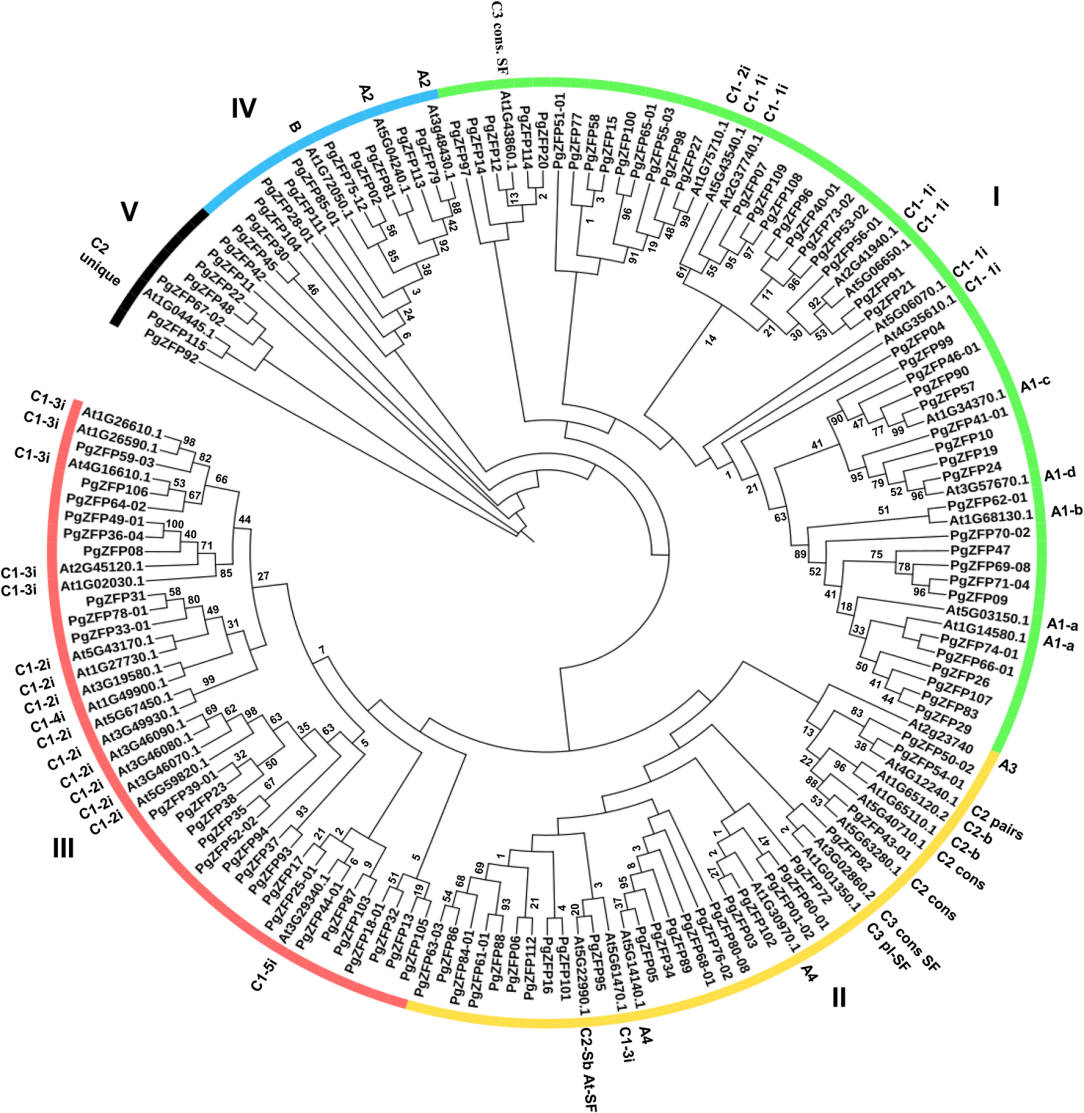
Phylogeny of the *PgZFP* gene family. The phylogenetic tree of the *PgZFP* gene family was constructed using all 115 *PgZFP* genes and the C2H2-ZFP genes of *Arabidopsis thaliana* (*At*) as outgroup. The subfamilies of the *PgZFP* gene family are indicated by I, II, III, IV, and V*.* The phylogenetic tree was constructed using the maximum-likelihood (ML) method with 1,000 bootstrap replications. The number nearby each cluster indicates the bootstrap confidence of the cluster in percentage (%).

To determine the origin and phylogeny of the *PgZFP* gene family in Jilin ginseng (Supplemental Fig. S1A), the putative proteins of the longest transcripts of all 115 *PgZFP* genes were used for the experiment (Supplemental Table S4). Forty-five representative *A. thaliana* (*At*) C2H2-ZFP genes selected from their phylogenetic tree (Supplemental Table S6) were used as outgroup (Supplemental Fig. S1B)^33^. The *PgZFP* genes were classified, with the AtC2H2-ZFP genes, into five clusters, defined I through V (Fig. 2). Cluster I included 26 *PgZFP* genes grouped with the members from the C1-2i, C1-3i, C1-4i, and C1-5i clusters of the AtC2H2-ZFP genes. Cluster II consisted of 25 *PgZFP* genes grouped with the members from the C2, C3, A3, and A4 subfamilies of the AtC2H2-ZFP genes. Cluster III was made of 46 *PgZFP* genes grouped with the members from the C1-1i, C1-2i, A1-a, A1-b, and A1-c clusters of the AtC2H2-ZFP genes. Cluster IV contained 11 *PgZFP* genes grouped with the members from the AtC2H2-ZFP A2 subfamily and B family. Cluster V was constituted of 7 *PgZFP* genes grouped with the only member from the AtC2H2-ZFP C2 subfamily^17^. These results suggested the ancient origin and diversity of the *PgZFP* gene family.

**Figure 3:**
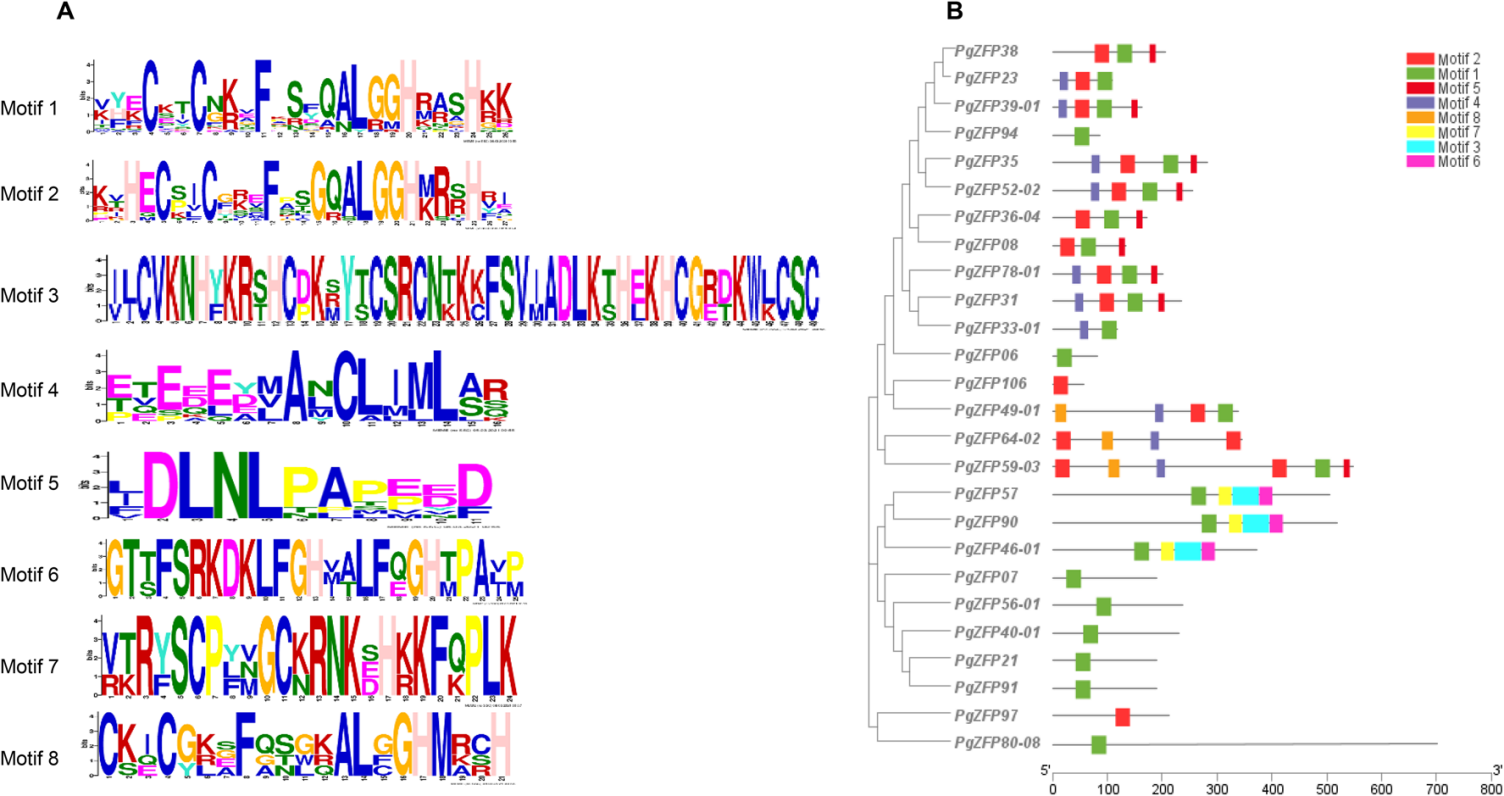
The conserved motifs and their distribution in the *PgZFP* genes. (**A**) Conserved motifs of *PgZFP* proteins. (**B**) Conserved motif distribution in the *PgZFP* proteins. The phylogenetic tree of the *PgZFP* proteins were constructed using the maximum-likelihood (ML) method.

Moreover, we analyzed the conserved motifs of the *PgZFP* gene family using the putative proteins of 26 representative *PgZFP* genes that have complete conserved domains within ORFs with criteria of motif length = 10–50 amino acids and a maximum motif number of 6, 8, or 10. When a maximum motif number of 6 was used, six conservative motifs were identified. When a maximum motif number of 8 was employed, eight conservative motifs were identified. When a maximum motif number of 10 was applied, ten conservative motifs were identified. Nevertheless, the six or eight motifs of the *PgZFP* genes identified with a maximum motif number of 6 or 8 were completely consistent with the first six or eight of the 10 motifs of the genes identified with a maximum motif number of 10. Figure 3A shows the eight conserved motifs of the *PgZFP g*enes identified with the maximum motif number of 8, defined Motif 1 through Motif 8. Motif 1 was identified in 88.5% of the 26 *PgZFP* genes examined (Fig. 3B), indicating the evolutionary conservation of the *PgZFP* gene family. The QALGGH of Motifs 1 and 2 plays important roles in DNA binding^8^. The EXEXXAXCLXXL (L-box) of Motif 4 is a leucine rich region that is considered to play an important role in protein–protein interactions^23^. Motif 5, as EAR domain, includes the core DLNL sequence and has been proven to play an important role in transcriptional inhibition and abiotic stress response^29,45^.

### Functional divergence of the *PgZFP* genes

**Figure 4:**
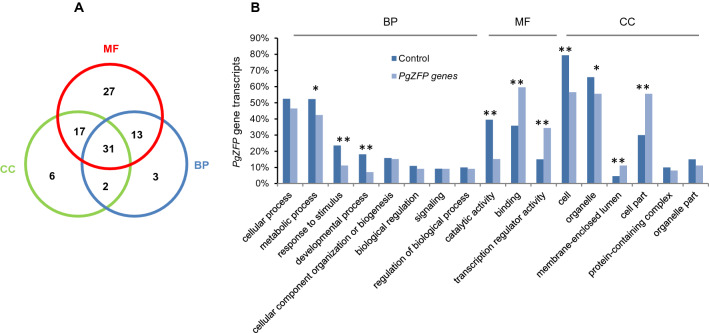
(**A**) GO categorization of the *PgZFP* gene transcripts. (**B**) Enrichment of the *PgZFP* gene transcripts. BP, Biological Process; MF, Molecular Function; CC, Cellular Component.

First, we categorized the *PgZFP* gene transcripts with gene ontology (GO). The 228 *PgZFP* gene transcripts were categorized into all three primary functional categories, MF (Molecular Function), BP (Biological Process), and CC (Cellular Component) (Fig. 4A). The MF category included 88 transcripts, of which 27 were MF-specific, 17 were categorized into both MF and CC categories, 13 were categorized into both MF and BP categories, and 31 were categorized into all three primary categories. The BP category included 49 transcripts, of which 3 were BP-specific, 2 were categorized into both BP and CC categories, and the remaining transcripts were categorized with MF, and with MF and CC as above. The CC category included 56 transcripts, of which 6 were CC-specific and the remaining transcripts were categorized with MF, with BP, and with MF and BP as above. The BP category was further categorized into eight subcategories at level 2, of which three were down-enriched in number of the *PgZFP* genes (*P* ≤ 0.05 or 0.01) (Fig. 4B). The MF category was categorized into three subcategories, catalytic activity, binding, and transcription regulator activity, of which the *PgZFP* genes involved in catalytic activity were down-enriched (*P* ≤ 0.01) and those involved in binding or transcription regulator activity were up-enriched (*P* ≤ 0.01). The CC category was categorized into six subcategories, of which two were down-enriched and two up-enriched in number of the *PgZFP* genes (*P* ≤ 0.05 or 0.01). These results suggested the functional differentiation, divergence, and specialty of the *PgZFP* genes.

Furthermore, we examined whether the GO categorization of the *PgZFP* gene family was consistent across tissues, developmental stages, and genotypes. The results showed that the *PgZFP* gene family was consistently categorized into the same 17 subcategories as above at level 2 across tissues, developmental stages, and genotypes (Supplemental Fig. S2). Nevertheless, the number of the *PgZFP* gene transcripts categorized into each of the 17 subcategories varied substantially across tissues, developmental stages, and genotypes.

Next, we analyzed the cis-regulatory elements of the *PgZFP* genes, such as promoter elements, because of their relationships with gene expression activities and potential biological functions. Since 73 of the 115 *PgZFP* genes were aligned to the Chinese ginseng genome^6^, the 1500-bp upstream sequences of the 73 *PgZFP* genes were searched for cis-regulatory elements. A total of 3709 cis-regulatory elements were identified for the 73 *PgZFP* genes and these elements were classified into 53 types, such as TATA-box, CAAT-box, SARE, ARE, AuxRE, and MBS. These elements are responsive to hormones, environmental stresses, and plant growth (Supplemental Fig. S3A). Of the 3709 cis-regulatory elements of the 73 *PgZFP* genes, 276 were responsive to hormones, including auxin, gibberellin, salicylic acid, abscisic acid, and MeJA (Supplemental Fig. S3B); 149 to environmental stresses, including defense, light, low-temperature, and drought (Supplemental Fig. S3C); and 82 to plant growth, such as seed, meristem expression, and endosperm expression (Supplemental Fig. S3D).

### Expression characteristics of the *PgZFP* gene transcripts in different tissues, at different developmental stages, and across genotypes

**Figure 5:**
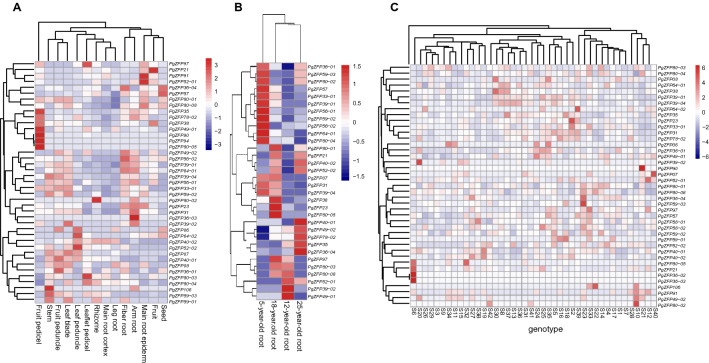
Expression heatmaps of the *PgZFP* gene transcripts in 14 tissues (**A**), four different aged roots (**B**) and the 4-year-old roots of 42 genotypes (**C**).

We characterized the expressions of the *PgZFP* gene family spatially, temperately, and across genotypes collected across the origin and diversity center of Jilin ginseng in different aspects. Analysis of a random selection of transcripts from the *PgZFP* gene family showed that the expressions of different gene transcripts varied dramatically in a tissue, at a developmental stage, or in a genotype. Nevertheless, the expression of a gene transcript was relatively consistent across tissues, developmental stages, and genotypes, even though its expression also varied across tissues, developmental stages, and genotypes (Supplemental Fig. S4). Of the 228 transcripts of the *PgZFP* gene family identified in this study, only 54.4–71.9% expressed in a single tissue (Supplemental Fig. S5A) and 27.6–33.8% expressed at a single developmental stage of root (Supplemental Fig. S5B). Forty-six of them expressed at all four developmental stages of the roots and 13, 5, 4, and 14 expressed specifically at 5-, 12-, 18-, and 25-year-old roots, respectively (Supplemental Fig. S5C). Among the genotypes studied, 49.1–64.5% of the *PgZFP* gene transcripts expressed in the 4-year-old plant root of a genotype (Supplemental Fig. S5D). Transcript expression heatmap analysis showed that the expressions of a vast majority of the transcripts in the *PgZFP* gene family was independently regulated across tissues, across developmental stages, and across genotypes (Fig. 5). Only *PgZFP94* and *PgZFP80-05* were found to be co-regulated across the tissues analyzed (Fig. 5A); none of the genes analyzed was co-regulated across the developmental stages of roots (Fig. 5B); and *PgZFP36-02* and *PgZFP36-03* were co-regulated across genotypes (Fig. 5C).

### Co-expression network of the *PgZFP* gene transcripts and its potential hub genes

**Figure 6:**
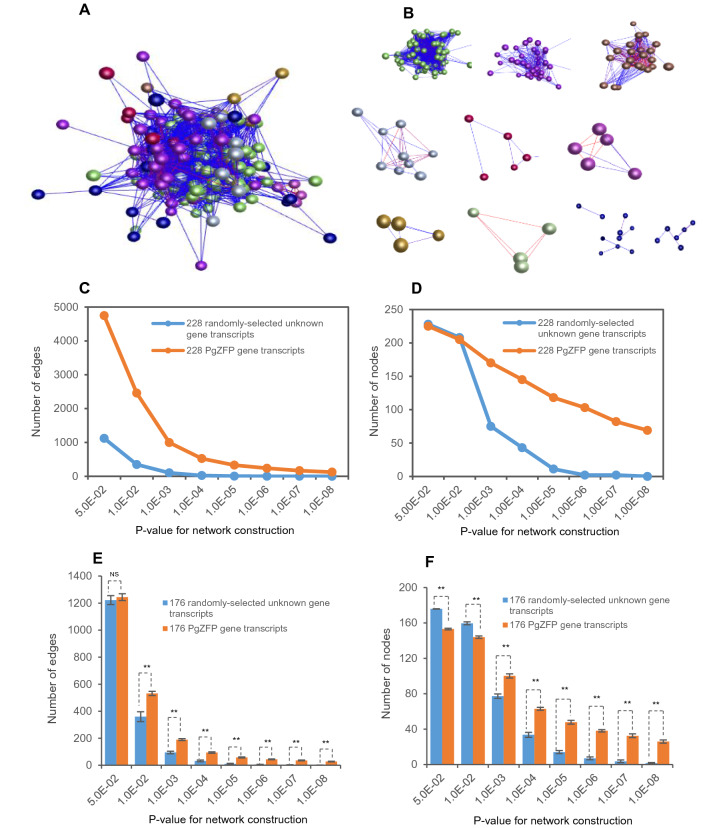
The co-expression network of the *PgZFP* gene transcripts expressed in the 4-year-old roots of 42 genotypes. (**A**) The co-expression network of the 228 *PgZFP* gene transcripts constructed at *P* ≤ 5.0E−02. It contains 228 gene transcript nodes and 4745 interaction edges. (**B**) The 12 clusters of the network. (**C, D**) Tendency of network formation at different *P* values: variation in numbers of nodes (**C**) and edges (**D**). (**E**,**F**) Statistics of the network formation tendency in numbers of edges (**E**) and nodes (**F**), with 20 replications. “**” indicates that the difference is significant at *P* ≤ 0.01; and “NS” indicates the difference is not significant at *P* ≤ 0.05. The randomly selected ginseng unknown gene transcripts were selected from Database A as controls.

The above phylogenetic analysis, GO categorization, and cis-regulatory element examination of the *PgZFP* gene family indicated that the gene family has substantially differentiated in sequence and functionality. The question is whether any relationship remains among the genes of the *PgZFP* gene family. Therefore, we conducted co-expression network analysis with *PgZFP* gene transcripts. The results showed that all 228 transcripts of the *PgZFP* gene family formed a single strong co-expression network (Fig. 6A). The network consisted of 228 nodes and 4745 edges that were clustered into eight clusters (Fig. 6B). In comparison, the network of the *PgZFP* gene family was much more robust than that of randomly selected ginseng unknown transcripts (Fig. 6C,D). Statistics showed that the *PgZFP* gene transcripts were more likely to form a co-expression network than the randomly selected ginseng unknown transcripts (Fig. 6E,F). These results suggested that although the gene family has substantially differentiated in sequence and functionality, the expression activities of its genes still maintain correlated, indicating their functional correlation. Further analysis revealed that eight of the 115 *PgZFP* genes, *PgZFP79*, *PgZFP82*, *PgZFP114*, *PgZFP87*, *PgZFP01-02*, *PgZFP48*, *PgZFP63-04*, and *PgZFP30*, likely played central roles in the network when *P* ≤ 0.001 was applied; therefore, these genes are likely the hub genes, with each gene having a connectivity of 30–43 (Supplemental Fig. S6). Moreover, we attempted to align these eight *PgZFP* genes to the Arabidopsis genome, but only *PgZFP79* and its paralogous gene, *PgZFP79P*, were aligned to the At3G48430 (*REF6*) in the Arabidopsis genome (see Fig. 1). This gene was found to be a positive regulator of flowering in an FLC-dependent pathway in Arabidopsis^46^.

### Response of the *PgZFP* genes to salt stress in ginseng

**Figure 7:**
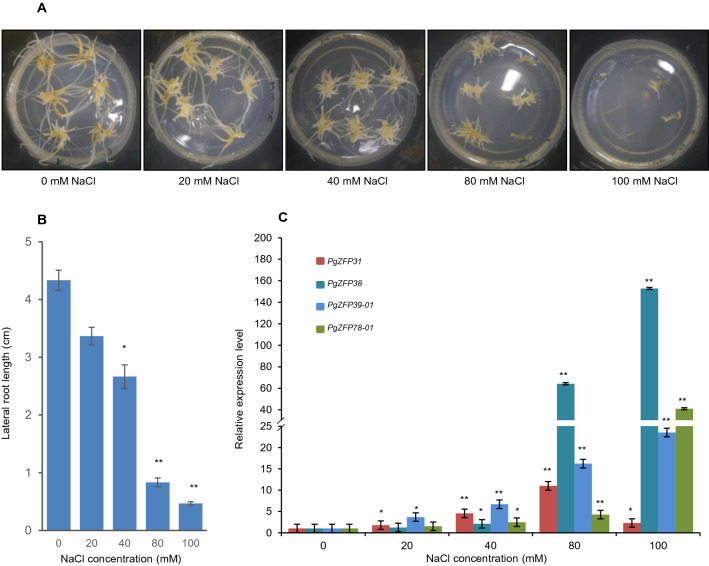
The responses of the *PgZFP* gene family to salt stress. (**A**) Ginseng adventitious roots stressed with different concentrations of NaCl on the B5 medium for 30 days. (**B**) Variation of the lateral root length of the salt-stressed roots. (**C**) The relative expressions of the *PgZFP* genes selected from the gene family in the salt-stressed roots. The expressions of the genes in the roots stressed with salt were presented as the expression level relative to those of the genes in the roots without salt stress (0 mM NaCl) considered as “1”. The *t*-test was used to test the mean difference of the gene expressions between control and salt-stressed roots. “*”, significant at *P* ≤ 0.05; “**”, significant at *P* ≤ 0.01.

Because the above cis-regulatory element analysis of the *PgZFP* gene family showed that the genes of the family are likely to be responsive to environmental stresses, we further studied the gene family in responses to environmental stresses, especially to salt stress. Sequence alignment analysis identified four *PgZFP* genes that were most well aligned to the Arabidopsis salt-responsive *STZ* and *AZF* genes, including *PgZFP31, PgZFP78-01, PgZFP38,* and *PgZFP39-01*. Of the four genes, *PgZFP31* was aligned to the Chinese ginseng genome^6^ and subjected to cis-regulatory element analysis, showing its potential responsiveness to hormones, environmental stresses, and growth. These four *PgZFP* genes were all from Cluster III of the family tree. Ginseng adventitious roots were used for the experiment. Figure 7A,B shows that the ginseng adventitious roots were sensitive to salt stress (NaCl). When the concentration of salt increased to 40 mM, the growth of ginseng roots was significantly inhibited (Fig. 7A), indicated by shorter roots (*P* ≤ 0.05) (Fig. 7B). The relative expression levels of all four genes were up-regulated by salt (Fig. 7C). When the concentration of the salt approached 20 mM NaCl, the relative expressions of two of the four genes started to increase significantly. When the concentration of the salt approached 40 mM NaCl or higher, the relative expressions of all four genes increased significantly (*P* ≤ 0.05) or extremely significantly (*P* ≤ 0.01), suggesting that at least four genes in the *PgZFP* gene family are involved in plant response to salt stress.

## Discussion

The C2H2-ZFP gene family has been shown in several species, including Arabidopsis, rice, tomato, and soybean, to play important functions in plant responses to abiotic and biotic stresses, plant growth and development, and hormone signal transduction^8–11,13–16^. However, the gene family remains unknown in ginseng. This study has, for the first time, genome-wide identified and characterized the gene family in ginseng. A total of 228 C2H2-ZFP gene transcripts, alternatively spliced from 115 C2H2-ZFP genes, are identified and defined *PgZFP* genes. Therefore, the *PgZFP* gene family consists of at least 115 gene members. This size of the gene family in ginseng is comparable with those of the gene family in poplar (109)^19^ and tomato (99)^47^, but it is smaller than those of the gene family in Arabidopsis (176)^17^, rice (189)^18^, *Medicago truncatula* (218)^20^, maize (211)^21^, and soybean (321)^22^. This result indicates that the *PgZFP* gene family is a moderate gene family. Nevertheless, this number of the *PgZFP* genes was identified in the Chinese ginseng cv. Damaya. Pan-transcriptome analysis reveals the number of genes in the family varies substantially among genotypes of *P.* ginseng, with pan-transcriptome of 149 *PgZFP* genes and a core-transcriptome of only 12 genes, suggesting that ginseng has a dispensable transcriptome varying by at least 137 *PgZFP* genes.

The *PgZFP* gene family is distributed in all 24 chromosomes of the Chinese ginseng genome, but only 17 (23%) of its 73 mapped *PgZFP* genes are syntenic to 11 of the Arabidopsis C2H2-ZFP genes. Analysis of 46 of the 115 *PgZFP* genes that have complete CDS shows that 40 (87%) of them were duplicated in the period of 18–41 MYA, suggesting that gene duplication plays a major role in the gene family expansion. The Ka/Ks ratio analysis indicates that purifying and neutral selections drive the family evolution. The present *PgZFP* gene family is classified into five clusters along with the C2H2-ZFP genes from Arabidopsis, suggesting that the gene family is an ancient gene family that originated before splitting between ginseng and Arabidopsis. Each cluster of the *PgZFP* gene family has specific conserved motifs distinguishing from other clusters; nevertheless, most genes of the gene family contain the highly conserved QALGGH motif or its variant, such as R/KALGGH. A previous study showed that the change of any amino acid in QALGGH may affect its DNA binding ability, and the mutation of the Q amino acid greatly reduced its DNA binding ability^48^. The C2H2-ZFP genes containing both QALGGH and I/D/FLN motifs played important roles in plant response to biotic and abiotic stresses^23^.

The *PgZFP* gene family was categorized into 17 subcategories at Level 2 and revealed to have 53 types of cis-regulatory elements responsive to multiple biological processes, suggesting that during the evolutionary process, changes have substantially occurred in the structure and upstream regions of *PgZFP* genes and these changes have affected their functional diversity^36–38^. The functional diversity of the gene family is larger than those of *PgbHLH*^49^, *PgNAC*^50^, and *PgGRAS*^51^ gene families in ginseng that were categorized into 11, 8, and 15 subcategories, respectively. Of the 228 *PgZFP* gene transcripts, 134 (59%) have binding functions. In comparison, poplar has also most (106) of the C2H2-ZFP genes involved in binding^19^ and tomato has all annotated C2H2-ZFP genes involved in binding, including nuclear acid binding, organic cyclic compound binding, and heterocyclic compound binding^47^. This suggests that the *PgZFP* genes play a role in transcriptional regulation by binding to downstream target genes^28,29,52^.

The expression analyses of the *PgZFP* gene family have resulted in several interesting findings. First, most of the genes in the *PgZFP* gene family expressed at a relatively low level in a tissue, at a developmental stage, and in the root of a genotype. The *PgZFP* genes that actively expressed in one tissue, at one developmental stage, and in the root of one genotype also tended to actively express in other tissues, at other developmental stages, and in the roots of other genotypes. Second, it is apparent that the expression relationships of the genes in the family are not consistent with their phylogenetic relationships determined by amino acid sequence similarity, suggesting that the genes having similar sequences may not have similar expression patterns. Third, the expressions of the transcripts spliced from the same gene may be substantially different in a tissue, at a developmental stage, and in the root of a genotype. Finally, of the 115 genes in the *PgZFP* gene family the expressions of only a few are co-regulated, while the expressions of a vast majority are independently regulated. Nevertheless, the genes of the *PgZFP* gene family are more likely to form a co-expression interaction network, of which some play central roles in the network, indicating that the gene members of the gene family functionally remain correlated^49^.

Previous studies showed that the C2H2-ZFP gene family plays important roles in growth and development, and plant responses to hormones and biotic and abiotic stresses^23^. The cis-regulatory element analysis of the *PgZFP* genes in the present study provides another line of evidence on these roles of the genes in ginseng. Moreover, four genes of the *PgZFP* gene family, *PgZFP31*, *PgZFP78-01*, *PgZFP38*, and *PgZFP39-01*, have been identified that were involved in response to salt stress, suggesting that the *PgZFP* gene family indeed plays roles in plant responses to abiotic stresses, particularly to salt stress in ginseng. Interestingly, the four *PgZFP* genes are all from Cluster III of the gene family tree. Of the four salt-stress responsive *PgZFP* genes examined, *PgZFP31* and *PgZFP78-01* have similar zinc finger structures to *STZ* that is involved in plant response to salt stress in Arabidopsis^14^. Both *PgZFP31* and *PgZFP78-01* belong to C1-2i type zinc finger proteins, have a high similarity in the DLN motif, and contain the FDLNI/L motif. The similar result has been obtained for *PgZFP38* and *PgZFP39-01* in comparison with *AZF1* that is also involved in plant response to salt stress in Arabidopsis^14,53^. These four *PgZFP* genes, therefore, provide gene resources for salt tolerance research and genetic improvement in ginseng.

## Conclusion

The *PgZFP* gene family is an ancient gene family consisting of approximately 115 *PgZFP* genes distributed among all 24 chromosomes of the ginseng genome. It originated before splitting of ginseng from Arabidopsis and its genes have substantially diverged in amino acid sequences and functionality, since they duplicated 18–41 MYA. Nevertheless, conserved motifs exist among the putative proteins of the genes in the family. Different gene members in the gene family express differently in a tissue, at a developmental stage, and in a genotype and the expression of the same gene varies across tissues, developmental stages, and genotypes, which further indicates differentiation of their functionality. Nevertheless, the genes in the family tend to express correlatively, forming a co-expression network, suggesting their functional correlation. Biologically, the *PgZFP* gene family plays important roles in plant response to salt stress in ginseng, from which four *PgZFP* genes are identified to be involved in response to salt stress in ginseng.

## Supplementary Information

Supplementary Figure S1.

Supplementary Figure S2.

Supplementary Figure S3.

Supplementary Figure S4.

Supplementary Figure S5.

Supplementary Figure S6.

Supplementary Table S1.

Supplementary Table S2.

Supplementary Table S3.

Supplementary Table S4.

Supplementary Table S5.

Supplementary Table S6.

## Data Availability

The data used for this study have been deposited at Sequence Read Archive (SRA) of National Center for Biotechnology Information (NCBI), BioProject PRJNA302556; and at Gene Expression Omnibus (GEO) of NCBI, SRP066368 and SRR13131364-SRR13131405. The plant materials are available from the corresponding authors, upon request.
