# Supplementary material for: Genome-wide analysis of the C2H2 zinc finger protein gene family and its response to salt stress in ginseng, Panax ginseng Meyer
Source: Sci Rep. 2022 Jun 17;12:10165. doi: 10.1038/s41598-022-14357-w (PMC9206012; doi:10.1038/s41598-022-14357-w)
Supplement: Supplementary file 1 — Supplementary Figure S1. [file 41598_2022_14357_MOESM1_ESM.pptx]

## Slide 1
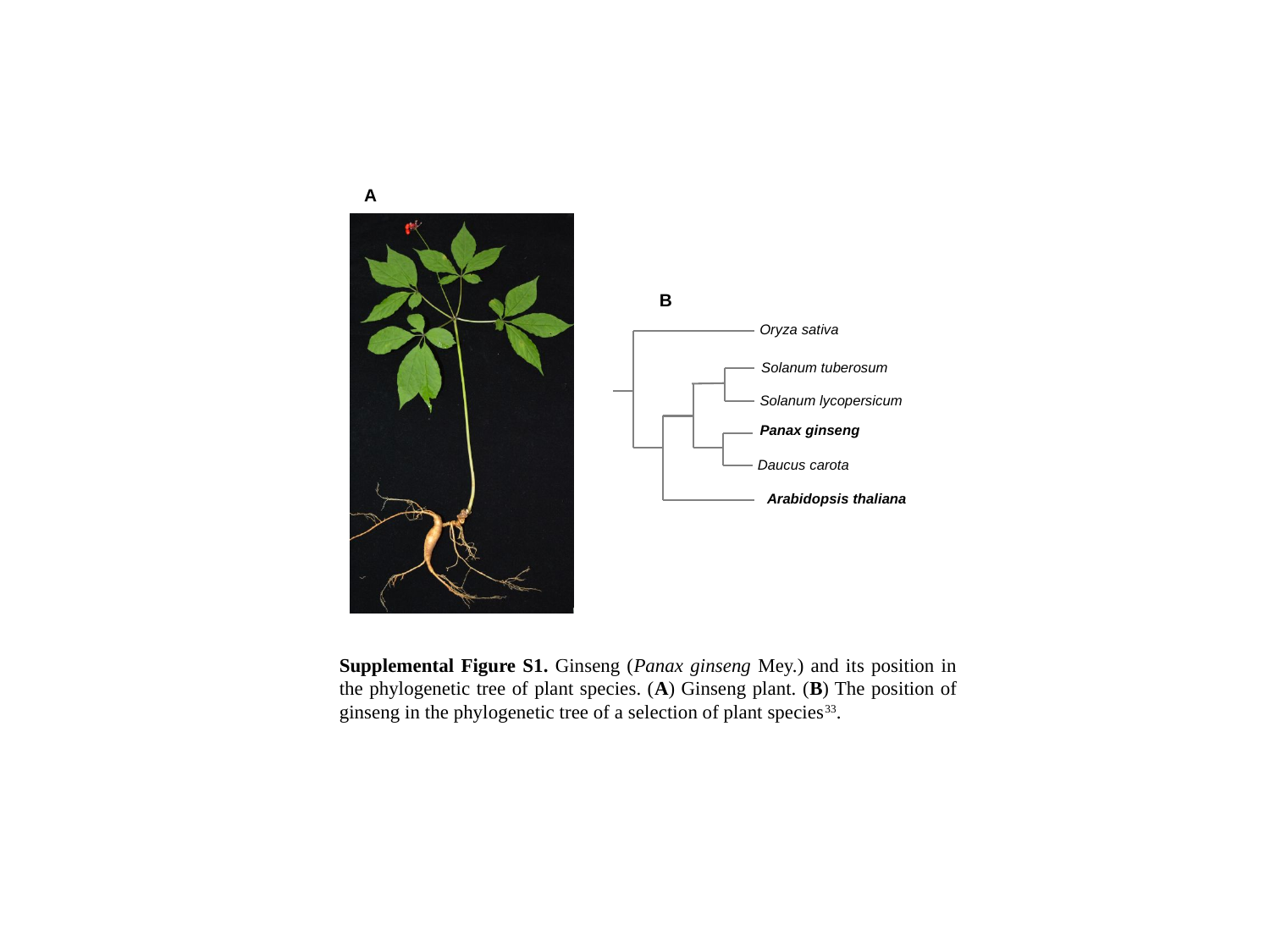

A
B
Oryza sativa
Solanum tuberosum
Solanum lycopersicum
Panax ginseng
Daucus carota
Arabidopsis thaliana
Supplemental Figure S1. Ginseng (Panax ginseng Mey.) and its position in the phylogenetic tree of plant species. (A) Ginseng plant. (B) The position of ginseng in the phylogenetic tree of a selection of plant species33.
