# Supplementary material for: Genome-wide analysis of the C2H2 zinc finger protein gene family and its response to salt stress in ginseng, Panax ginseng Meyer
Source: Sci Rep. 2022 Jun 17;12:10165. doi: 10.1038/s41598-022-14357-w (PMC9206012; doi:10.1038/s41598-022-14357-w)
Supplement: Supplementary file 2 — Supplementary Figure S2. [file 41598_2022_14357_MOESM2_ESM.pptx]

## Slide 1
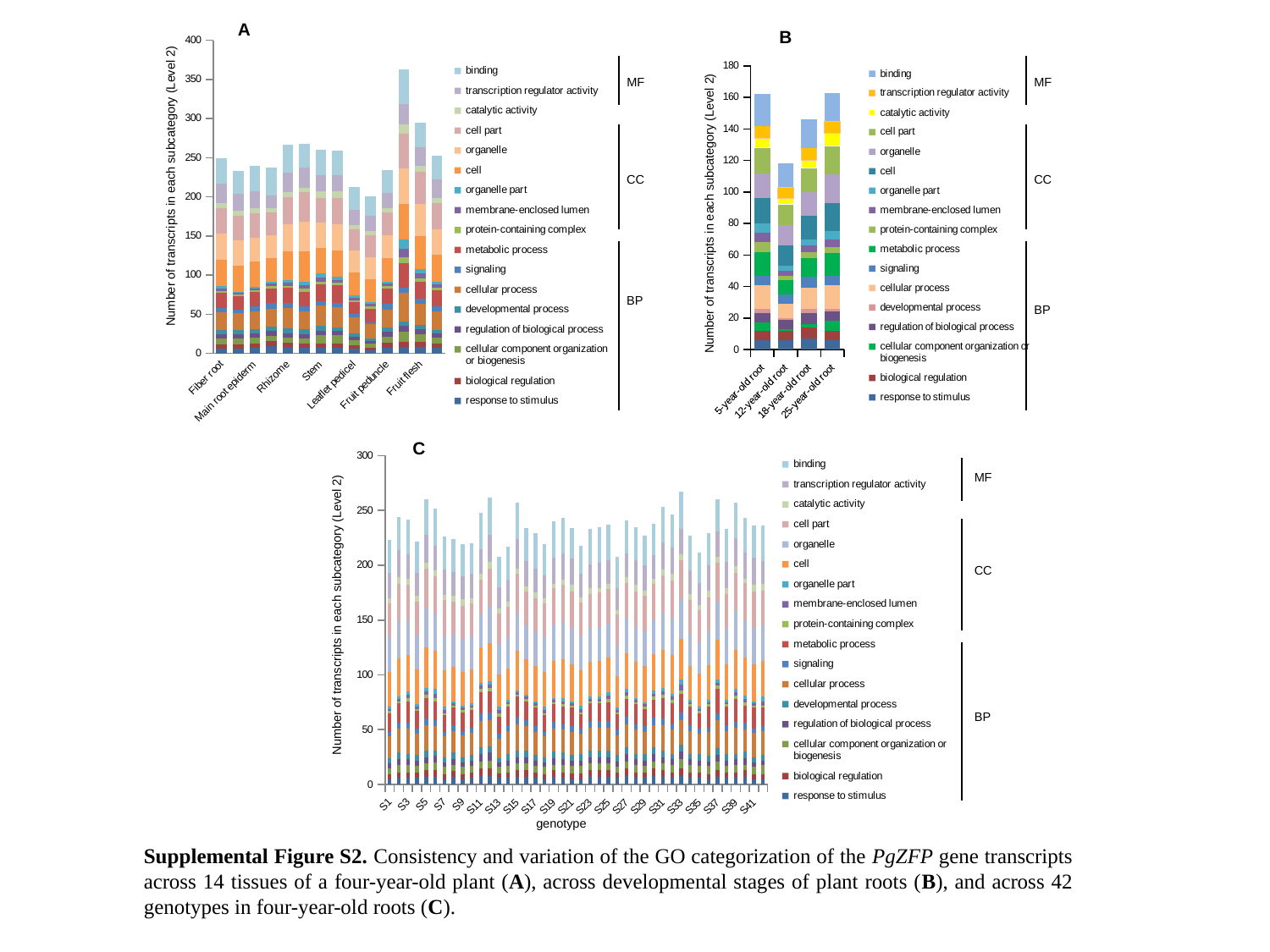

A
B
### Chart
| Category | response to stimulus | biological regulation | cellular component organization or biogenesis | regulation of biological process | developmental process | cellular process | signaling | metabolic process | protein-containing complex | membrane-enclosed lumen | organelle part | cell | organelle | cell part | catalytic activity | transcription regulator activity | binding |
|---|---|---|---|---|---|---|---|---|---|---|---|---|---|---|---|---|---|
| Fiber root | 6.0 | 6.0 | 7.0 | 5.0 | 6.0 | 23.0 | 5.0 | 19.0 | 3.0 | 3.0 | 3.0 | 34.0 | 33.0 | 33.0 | 6.0 | 25.0 | 32.0 |
| Leg root | 6.0 | 6.0 | 7.0 | 5.0 | 6.0 | 21.0 | 5.0 | 17.0 | 2.0 | 2.0 | 2.0 | 33.0 | 32.0 | 32.0 | 6.0 | 22.0 | 29.0 |
| Main root epiderm | 7.0 | 6.0 | 7.0 | 6.0 | 5.0 | 23.0 | 6.0 | 19.0 | 2.0 | 2.0 | 2.0 | 32.0 | 31.0 | 31.0 | 6.0 | 22.0 | 33.0 |
| Main root cortex | 9.0 | 7.0 | 6.0 | 7.0 | 5.0 | 23.0 | 7.0 | 19.0 | 3.0 | 3.0 | 3.0 | 30.0 | 29.0 | 29.0 | 5.0 | 17.0 | 35.0 |
| Rhizome | 8.0 | 6.0 | 6.0 | 6.0 | 6.0 | 26.0 | 6.0 | 20.0 | 2.0 | 4.0 | 4.0 | 36.0 | 35.0 | 35.0 | 6.0 | 25.0 | 36.0 |
| Arm root | 7.0 | 6.0 | 6.0 | 6.0 | 6.0 | 23.0 | 6.0 | 19.0 | 4.0 | 4.0 | 4.0 | 39.0 | 38.0 | 38.0 | 6.0 | 25.0 | 31.0 |
| Stem | 7.0 | 6.0 | 10.0 | 6.0 | 6.0 | 26.0 | 6.0 | 21.0 | 4.0 | 5.0 | 5.0 | 33.0 | 32.0 | 32.0 | 8.0 | 21.0 | 32.0 |
| Leaf peduncle | 7.0 | 6.0 | 10.0 | 6.0 | 4.0 | 26.0 | 6.0 | 22.0 | 3.0 | 4.0 | 4.0 | 34.0 | 33.0 | 33.0 | 9.0 | 21.0 | 31.0 |
| Leaflet pedicel | 6.0 | 4.0 | 7.0 | 4.0 | 5.0 | 20.0 | 4.0 | 16.0 | 2.0 | 3.0 | 3.0 | 29.0 | 28.0 | 28.0 | 5.0 | 19.0 | 30.0 |
| Leaf peduncle | 4.0 | 3.0 | 6.0 | 3.0 | 3.0 | 18.0 | 3.0 | 17.0 | 3.0 | 3.0 | 3.0 | 29.0 | 28.0 | 28.0 | 5.0 | 20.0 | 25.0 |
| Fruit peduncle | 7.0 | 7.0 | 7.0 | 7.0 | 5.0 | 23.0 | 7.0 | 20.0 | 3.0 | 3.0 | 3.0 | 30.0 | 29.0 | 29.0 | 6.0 | 19.0 | 29.0 |
| Fruit pedicel | 8.0 | 7.0 | 13.0 | 7.0 | 6.0 | 36.0 | 7.0 | 31.0 | 8.0 | 11.0 | 11.0 | 46.0 | 45.0 | 45.0 | 12.0 | 25.0 | 45.0 |
| Fruit flesh | 8.0 | 7.0 | 9.0 | 7.0 | 5.0 | 27.0 | 7.0 | 22.0 | 4.0 | 6.0 | 6.0 | 42.0 | 41.0 | 41.0 | 8.0 | 23.0 | 32.0 |
| Seed | 7.0 | 6.0 | 7.0 | 6.0 | 4.0 | 24.0 | 6.0 | 21.0 | 3.0 | 4.0 | 4.0 | 34.0 | 33.0 | 33.0 | 7.0 | 23.0 | 31.0 |Number of transcripts in each subcategory (Level 2)
MF
CC
BP
### Chart
| Category | response to stimulus | biological regulation | cellular component organization or biogenesis | regulation of biological process | developmental process | cellular process | signaling | metabolic process | protein-containing complex | membrane-enclosed lumen | organelle part | cell | organelle | cell part | catalytic activity | transcription regulator activity | binding |
|---|---|---|---|---|---|---|---|---|---|---|---|---|---|---|---|---|---|
| 5-year-old root | 6.0 | 6.0 | 5.0 | 6.0 | 3.0 | 15.0 | 6.0 | 15.0 | 6.0 | 6.0 | 6.0 | 16.0 | 16.0 | 16.0 | 6.0 | 8.0 | 20.0 |
| 12-year-old root | 6.0 | 6.0 | 1.0 | 6.0 | 1.0 | 9.0 | 6.0 | 9.0 | 3.0 | 3.0 | 3.0 | 13.0 | 13.0 | 13.0 | 4.0 | 7.0 | 15.0 |
| 18-year-old root | 7.0 | 7.0 | 2.0 | 7.0 | 3.0 | 13.0 | 7.0 | 12.0 | 4.0 | 4.0 | 4.0 | 15.0 | 15.0 | 15.0 | 5.0 | 8.0 | 18.0 |
| 25-year-old root | 6.0 | 6.0 | 6.0 | 6.0 | 2.0 | 15.0 | 6.0 | 14.0 | 4.0 | 5.0 | 5.0 | 18.0 | 18.0 | 18.0 | 8.0 | 8.0 | 18.0 |Number of transcripts in each subcategory (Level 2)
MF
CC
BP
### Chart
| Category | response to stimulus | biological regulation | cellular component organization or biogenesis | regulation of biological process | developmental process | cellular process | signaling | metabolic process | protein-containing complex | membrane-enclosed lumen | organelle part | cell | organelle | cell part | catalytic activity | transcription regulator activity | binding |
|---|---|---|---|---|---|---|---|---|---|---|---|---|---|---|---|---|---|
| S1 | 5.0 | 4.0 | 6.0 | 4.0 | 5.0 | 20.0 | 4.0 | 17.0 | 2.0 | 2.0 | 2.0 | 32.0 | 31.0 | 31.0 | 5.0 | 23.0 | 30.0 |
| S2 | 6.0 | 5.0 | 7.0 | 5.0 | 6.0 | 22.0 | 5.0 | 18.0 | 2.0 | 2.0 | 2.0 | 35.0 | 34.0 | 34.0 | 6.0 | 25.0 | 30.0 |
| S3 | 6.0 | 5.0 | 7.0 | 5.0 | 5.0 | 23.0 | 5.0 | 20.0 | 3.0 | 3.0 | 3.0 | 33.0 | 32.0 | 32.0 | 6.0 | 22.0 | 32.0 |
| S4 | 6.0 | 5.0 | 6.0 | 5.0 | 5.0 | 19.0 | 5.0 | 16.0 | 2.0 | 2.0 | 2.0 | 32.0 | 31.0 | 31.0 | 5.0 | 21.0 | 29.0 |
| S5 | 7.0 | 6.0 | 6.0 | 6.0 | 6.0 | 23.0 | 6.0 | 19.0 | 3.0 | 3.0 | 3.0 | 37.0 | 36.0 | 36.0 | 5.0 | 26.0 | 32.0 |
| S6 | 7.0 | 6.0 | 7.0 | 6.0 | 5.0 | 22.0 | 6.0 | 17.0 | 3.0 | 4.0 | 4.0 | 35.0 | 34.0 | 34.0 | 5.0 | 23.0 | 34.0 |
| S7 | 5.0 | 4.0 | 7.0 | 4.0 | 5.0 | 19.0 | 4.0 | 15.0 | 2.0 | 3.0 | 3.0 | 33.0 | 32.0 | 32.0 | 5.0 | 23.0 | 30.0 |
| S8 | 7.0 | 5.0 | 6.0 | 5.0 | 6.0 | 20.0 | 5.0 | 16.0 | 2.0 | 2.0 | 2.0 | 31.0 | 30.0 | 30.0 | 5.0 | 22.0 | 30.0 |
| S9 | 5.0 | 4.0 | 7.0 | 4.0 | 5.0 | 20.0 | 4.0 | 17.0 | 2.0 | 2.0 | 2.0 | 31.0 | 30.0 | 30.0 | 6.0 | 21.0 | 29.0 |
| S10 | 6.0 | 5.0 | 6.0 | 5.0 | 5.0 | 20.0 | 5.0 | 16.0 | 2.0 | 2.0 | 2.0 | 31.0 | 30.0 | 30.0 | 5.0 | 22.0 | 28.0 |
| S11 | 8.0 | 7.0 | 6.0 | 7.0 | 6.0 | 24.0 | 7.0 | 19.0 | 3.0 | 3.0 | 3.0 | 32.0 | 31.0 | 31.0 | 5.0 | 23.0 | 33.0 |
| S12 | 8.0 | 7.0 | 7.0 | 7.0 | 6.0 | 24.0 | 7.0 | 19.0 | 3.0 | 3.0 | 3.0 | 35.0 | 34.0 | 34.0 | 6.0 | 25.0 | 34.0 |
| S13 | 6.0 | 4.0 | 6.0 | 4.0 | 4.0 | 18.0 | 4.0 | 16.0 | 3.0 | 3.0 | 3.0 | 29.0 | 28.0 | 28.0 | 5.0 | 19.0 | 28.0 |
| S14 | 6.0 | 5.0 | 6.0 | 5.0 | 6.0 | 21.0 | 5.0 | 17.0 | 2.0 | 2.0 | 2.0 | 29.0 | 28.0 | 28.0 | 5.0 | 20.0 | 30.0 |
| S15 | 7.0 | 6.0 | 6.0 | 6.0 | 6.0 | 24.0 | 6.0 | 19.0 | 2.0 | 2.0 | 2.0 | 36.0 | 35.0 | 35.0 | 5.0 | 27.0 | 33.0 |
| S16 | 7.0 | 6.0 | 6.0 | 6.0 | 6.0 | 22.0 | 6.0 | 17.0 | 2.0 | 2.0 | 2.0 | 32.0 | 31.0 | 31.0 | 5.0 | 23.0 | 30.0 |
| S17 | 6.0 | 5.0 | 6.0 | 5.0 | 5.0 | 21.0 | 5.0 | 17.0 | 2.0 | 2.0 | 2.0 | 32.0 | 31.0 | 31.0 | 5.0 | 22.0 | 32.0 |
| S18 | 5.0 | 4.0 | 7.0 | 4.0 | 5.0 | 19.0 | 4.0 | 15.0 | 2.0 | 3.0 | 3.0 | 32.0 | 31.0 | 31.0 | 5.0 | 21.0 | 28.0 |
| S19 | 7.0 | 6.0 | 5.0 | 6.0 | 6.0 | 21.0 | 6.0 | 16.0 | 2.0 | 2.0 | 2.0 | 34.0 | 33.0 | 33.0 | 4.0 | 24.0 | 33.0 |
| S20 | 6.0 | 5.0 | 7.0 | 5.0 | 6.0 | 21.0 | 5.0 | 16.0 | 2.0 | 3.0 | 3.0 | 35.0 | 34.0 | 34.0 | 5.0 | 24.0 | 32.0 |
| S21 | 5.0 | 5.0 | 7.0 | 5.0 | 5.0 | 21.0 | 5.0 | 17.0 | 2.0 | 2.0 | 2.0 | 34.0 | 33.0 | 33.0 | 6.0 | 24.0 | 28.0 |
| S22 | 5.0 | 5.0 | 7.0 | 5.0 | 6.0 | 18.0 | 5.0 | 13.0 | 2.0 | 3.0 | 3.0 | 32.0 | 31.0 | 31.0 | 5.0 | 21.0 | 26.0 |
| S23 | 7.0 | 6.0 | 6.0 | 6.0 | 6.0 | 21.0 | 6.0 | 16.0 | 2.0 | 2.0 | 2.0 | 32.0 | 31.0 | 31.0 | 5.0 | 22.0 | 32.0 |
| S24 | 7.0 | 6.0 | 6.0 | 6.0 | 6.0 | 21.0 | 6.0 | 16.0 | 2.0 | 2.0 | 2.0 | 33.0 | 31.0 | 31.0 | 4.0 | 23.0 | 33.0 |
| S25 | 7.0 | 6.0 | 6.0 | 6.0 | 6.0 | 21.0 | 6.0 | 17.0 | 3.0 | 3.0 | 3.0 | 32.0 | 31.0 | 31.0 | 5.0 | 22.0 | 32.0 |
| S26 | 6.0 | 5.0 | 5.0 | 5.0 | 6.0 | 18.0 | 5.0 | 14.0 | 2.0 | 2.0 | 2.0 | 29.0 | 28.0 | 28.0 | 4.0 | 20.0 | 29.0 |
| S27 | 8.0 | 7.0 | 6.0 | 7.0 | 6.0 | 21.0 | 7.0 | 16.0 | 3.0 | 3.0 | 3.0 | 33.0 | 32.0 | 32.0 | 5.0 | 22.0 | 30.0 |
| S28 | 6.0 | 5.0 | 7.0 | 5.0 | 5.0 | 22.0 | 5.0 | 18.0 | 2.0 | 2.0 | 2.0 | 33.0 | 32.0 | 32.0 | 6.0 | 23.0 | 30.0 |
| S29 | 6.0 | 5.0 | 6.0 | 5.0 | 6.0 | 20.0 | 5.0 | 16.0 | 2.0 | 2.0 | 2.0 | 33.0 | 32.0 | 32.0 | 5.0 | 23.0 | 27.0 |
| S30 | 8.0 | 7.0 | 6.0 | 7.0 | 6.0 | 20.0 | 7.0 | 16.0 | 3.0 | 3.0 | 3.0 | 33.0 | 32.0 | 32.0 | 5.0 | 21.0 | 29.0 |
| S31 | 7.0 | 6.0 | 6.0 | 6.0 | 6.0 | 23.0 | 6.0 | 19.0 | 3.0 | 3.0 | 3.0 | 35.0 | 34.0 | 34.0 | 5.0 | 25.0 | 32.0 |
| S32 | 6.0 | 5.0 | 7.0 | 5.0 | 5.0 | 22.0 | 5.0 | 19.0 | 3.0 | 3.0 | 3.0 | 35.0 | 34.0 | 34.0 | 6.0 | 24.0 | 30.0 |
| S33 | 8.0 | 7.0 | 8.0 | 7.0 | 6.0 | 23.0 | 7.0 | 17.0 | 3.0 | 5.0 | 5.0 | 37.0 | 36.0 | 36.0 | 5.0 | 23.0 | 34.0 |
| S34 | 6.0 | 5.0 | 7.0 | 5.0 | 5.0 | 21.0 | 5.0 | 17.0 | 2.0 | 2.0 | 2.0 | 31.0 | 30.0 | 30.0 | 6.0 | 21.0 | 32.0 |
| S35 | 6.0 | 5.0 | 6.0 | 5.0 | 6.0 | 18.0 | 5.0 | 14.0 | 2.0 | 2.0 | 2.0 | 30.0 | 29.0 | 29.0 | 5.0 | 20.0 | 28.0 |
| S36 | 5.0 | 4.0 | 8.0 | 4.0 | 5.0 | 22.0 | 4.0 | 19.0 | 2.0 | 2.0 | 2.0 | 32.0 | 31.0 | 31.0 | 6.0 | 23.0 | 29.0 |
| S37 | 7.0 | 6.0 | 8.0 | 6.0 | 6.0 | 26.0 | 6.0 | 22.0 | 3.0 | 3.0 | 3.0 | 36.0 | 35.0 | 35.0 | 6.0 | 23.0 | 29.0 |
| S38 | 6.0 | 5.0 | 6.0 | 5.0 | 6.0 | 21.0 | 5.0 | 17.0 | 2.0 | 2.0 | 2.0 | 33.0 | 32.0 | 32.0 | 5.0 | 24.0 | 30.0 |
| S39 | 6.0 | 5.0 | 7.0 | 5.0 | 5.0 | 24.0 | 5.0 | 21.0 | 3.0 | 3.0 | 3.0 | 36.0 | 35.0 | 35.0 | 6.0 | 26.0 | 32.0 |
| S40 | 7.0 | 6.0 | 5.0 | 6.0 | 6.0 | 20.0 | 6.0 | 16.0 | 3.0 | 3.0 | 3.0 | 35.0 | 34.0 | 34.0 | 4.0 | 24.0 | 31.0 |
| S41 | 5.0 | 4.0 | 7.0 | 4.0 | 5.0 | 22.0 | 4.0 | 19.0 | 2.0 | 2.0 | 2.0 | 34.0 | 33.0 | 33.0 | 6.0 | 25.0 | 29.0 |
| S42 | 5.0 | 4.0 | 9.0 | 4.0 | 5.0 | 22.0 | 4.0 | 17.0 | 2.0 | 4.0 | 4.0 | 33.0 | 32.0 | 32.0 | 6.0 | 21.0 | 32.0 |MF
CC
Number of transcripts in each subcategory (Level 2)
BP
C
genotype
Supplemental Figure S2. Consistency and variation of the GO categorization of the PgZFP gene transcripts across 14 tissues of a four-year-old plant (A), across developmental stages of plant roots (B), and across 42 genotypes in four-year-old roots (C).
