# Supplementary material for: Genome-wide analysis of the C2H2 zinc finger protein gene family and its response to salt stress in ginseng, Panax ginseng Meyer
Source: Sci Rep. 2022 Jun 17;12:10165. doi: 10.1038/s41598-022-14357-w (PMC9206012; doi:10.1038/s41598-022-14357-w)
Supplement: Supplementary file 3 — Supplementary Figure S3. [file 41598_2022_14357_MOESM3_ESM.pptx]

## Slide 1
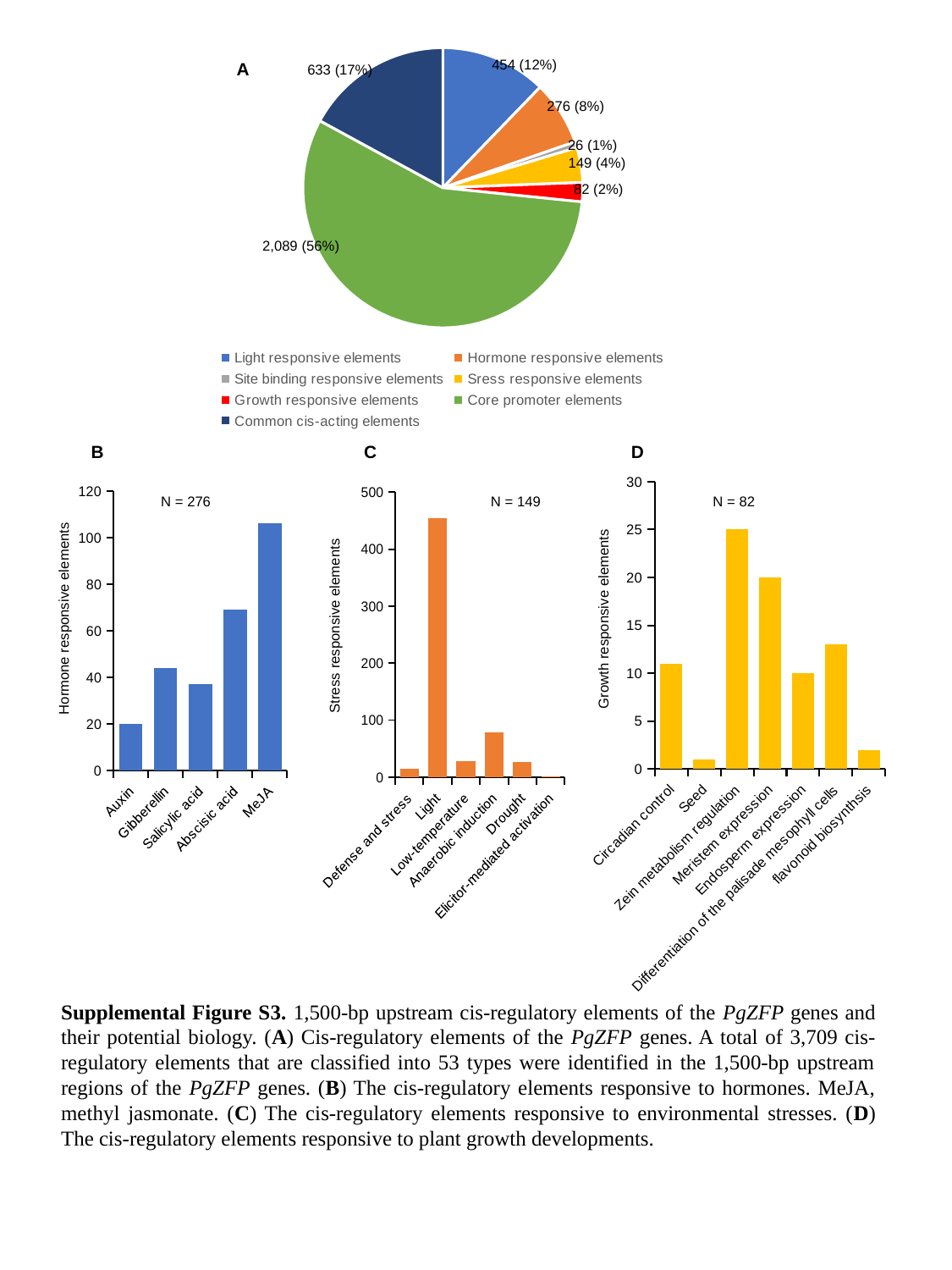

### Chart
| Category | |
|---|---|
| Light responsive elements | 454.0 |
| Hormone responsive elements | 276.0 |
| Site binding responsive elements | 26.0 |
| Sress responsive elements | 149.0 |
| Growth responsive elements | 82.0 |
| Core promoter elements | 2089.0 |
| Common cis-acting elements | 633.0 |454 (12%)
A
633 (17%)
276 (8%)
26 (1%)
149 (4%)
82 (2%)
2,089 (56%)
B
C
D
### Chart
| Category | |
|---|---|
| Circadian control | 11.0 |
| Seed | 1.0 |
| Zein metabolism regulation | 25.0 |
| Meristem expression | 20.0 |
| Endosperm expression | 10.0 |
| Differentiation of the palisade mesophyll cells | 13.0 |
| flavonoid biosynthsis | 2.0 |Growth responsive elements
### Chart
| Category | |
|---|---|
| Auxin | 20.0 |
| Gibberellin | 44.0 |
| Salicylic acid | 37.0 |
| Abscisic acid | 69.0 |
| MeJA | 106.0 |
### Chart
| Category | |
|---|---|
| Defense and stress | 14.0 |
| Light | 454.0 |
| Low-temperature | 28.0 |
| Anaerobic induction | 79.0 |
| Drought | 26.0 |
| Elicitor-mediated activation | 2.0 |Stress responsive elements
N = 276
N = 149
N = 82
Hormone responsive elements
Supplemental Figure S3. 1,500-bp upstream cis-regulatory elements of the PgZFP genes and their potential biology. (A) Cis-regulatory elements of the PgZFP genes. A total of 3,709 cis-regulatory elements that are classified into 53 types were identified in the 1,500-bp upstream regions of the PgZFP genes. (B) The cis-regulatory elements responsive to hormones. MeJA, methyl jasmonate. (C) The cis-regulatory elements responsive to environmental stresses. (D) The cis-regulatory elements responsive to plant growth developments.
