# Supplementary material for: Genome-wide analysis of the C2H2 zinc finger protein gene family and its response to salt stress in ginseng, Panax ginseng Meyer
Source: Sci Rep. 2022 Jun 17;12:10165. doi: 10.1038/s41598-022-14357-w (PMC9206012; doi:10.1038/s41598-022-14357-w)
Supplement: Supplementary file 4 — Supplementary Figure S4. [file 41598_2022_14357_MOESM4_ESM.pptx]

## Slide 1
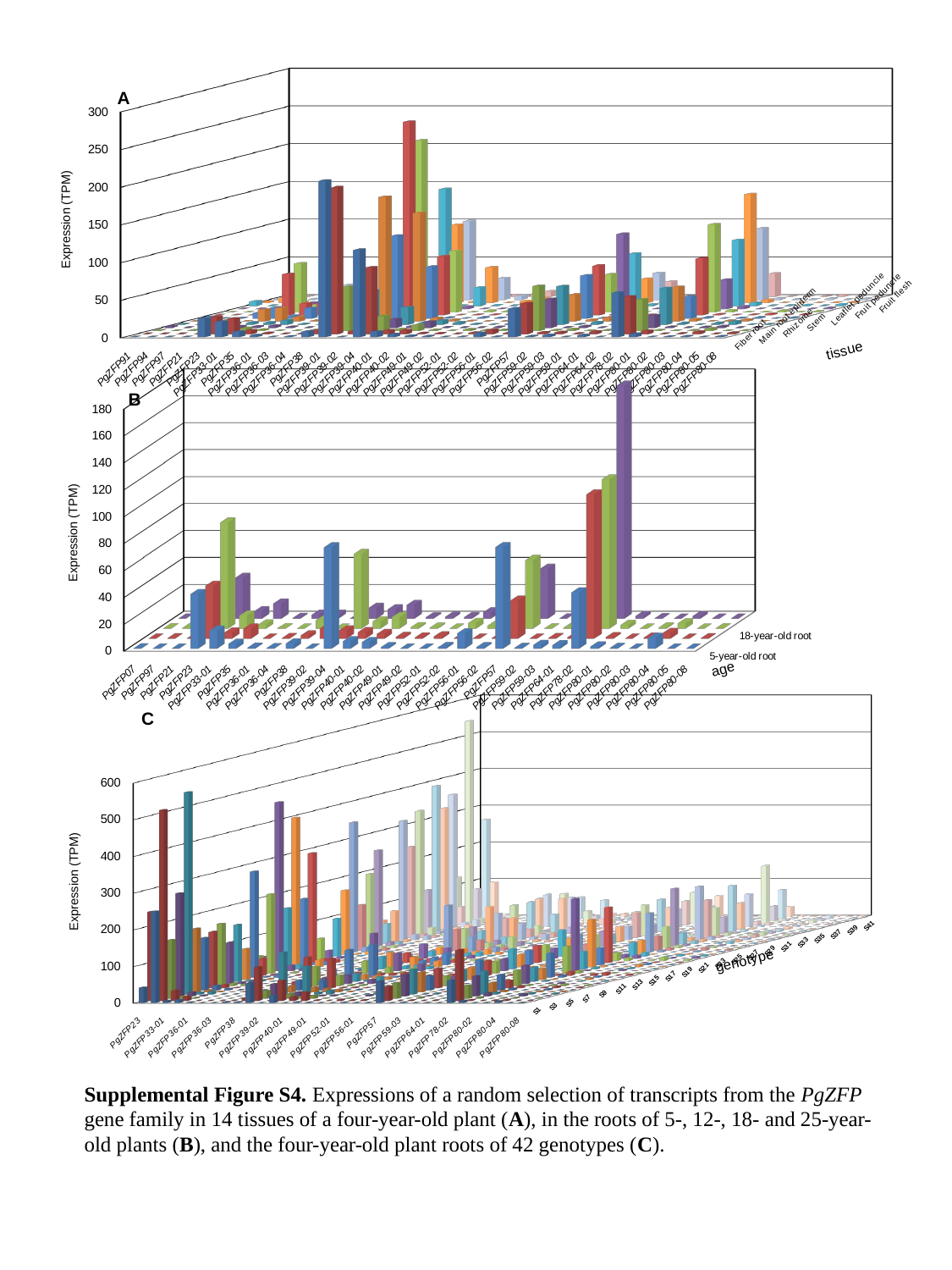

[unsupported chart]
A
Expression (TPM)
tissue
[unsupported chart]
Expression (TPM)
age
[unsupported chart]
Expression (TPM)
Supplemental Figure S4. Expressions of a random selection of transcripts from the PgZFP gene family in 14 tissues of a four-year-old plant (A), in the roots of 5-, 12-, 18- and 25-year-old plants (B), and the four-year-old plant roots of 42 genotypes (C).
