# Supplementary material for: Genome-wide analysis of the C2H2 zinc finger protein gene family and its response to salt stress in ginseng, Panax ginseng Meyer
Source: Sci Rep. 2022 Jun 17;12:10165. doi: 10.1038/s41598-022-14357-w (PMC9206012; doi:10.1038/s41598-022-14357-w)
Supplement: Supplementary file 5 — Supplementary Figure S5. [file 41598_2022_14357_MOESM5_ESM.pptx]

## Slide 1
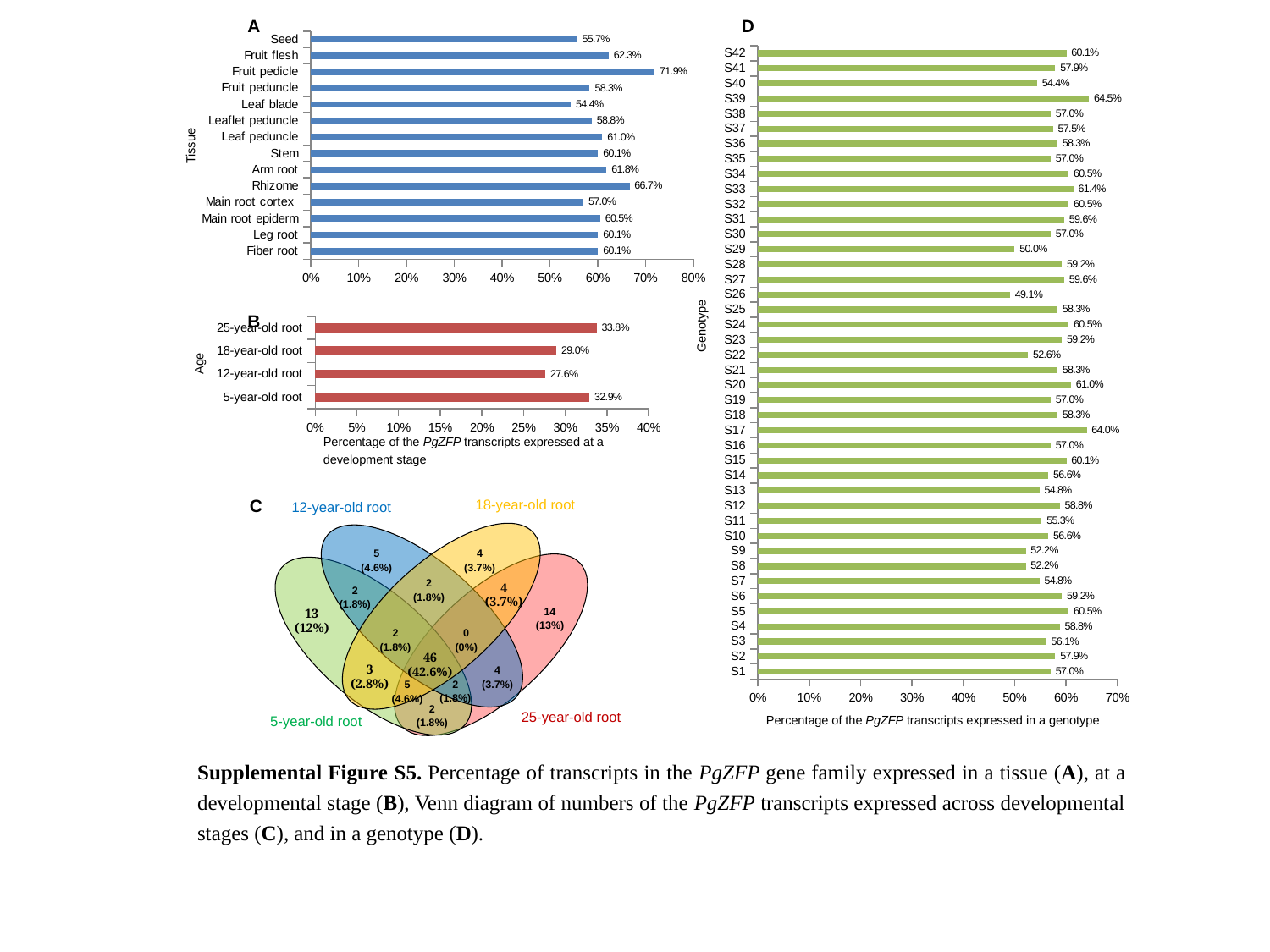

A
D
### Chart
| Category | |
|---|---|
| S1 | 0.5701754385964912 |
| S2 | 0.5789473684210527 |
| S3 | 0.5614035087719298 |
| S4 | 0.5877192982456141 |
| S5 | 0.6052631578947368 |
| S6 | 0.5921052631578947 |
| S7 | 0.5482456140350878 |
| S8 | 0.5219298245614035 |
| S9 | 0.5219298245614035 |
| S10 | 0.5657894736842105 |
| S11 | 0.5526315789473684 |
| S12 | 0.5877192982456141 |
| S13 | 0.5482456140350878 |
| S14 | 0.5657894736842105 |
| S15 | 0.6008771929824561 |
| S16 | 0.5701754385964912 |
| S17 | 0.6403508771929824 |
| S18 | 0.5833333333333333 |
| S19 | 0.5701754385964912 |
| S20 | 0.6096491228070176 |
| S21 | 0.5833333333333333 |
| S22 | 0.5263157894736843 |
| S23 | 0.5921052631578947 |
| S24 | 0.6052631578947368 |
| S25 | 0.5833333333333333 |
| S26 | 0.49122807017543857 |
| S27 | 0.5964912280701755 |
| S28 | 0.5921052631578947 |
| S29 | 0.5 |
| S30 | 0.5701754385964912 |
| S31 | 0.5964912280701755 |
| S32 | 0.6052631578947368 |
| S33 | 0.6140350877192983 |
| S34 | 0.6052631578947368 |
| S35 | 0.5701754385964912 |
| S36 | 0.5833333333333333 |
| S37 | 0.5745614035087719 |
| S38 | 0.5701754385964912 |
| S39 | 0.6447368421052632 |
| S40 | 0.543859649122807 |
| S41 | 0.5789473684210527 |
| S42 | 0.6008771929824561 |Percentage of the PgZFP transcripts expressed in a genotype
### Chart
| Category | |
|---|---|
| Fiber root | 0.6008771929824561 |
| Leg root | 0.6008771929824561 |
| Main root epiderm | 0.6052631578947368 |
| Main root cortex | 0.5701754385964912 |
| Rhizome | 0.6666666666666667 |
| Arm root | 0.618421052631579 |
| Stem | 0.6008771929824561 |
| Leaf peduncle | 0.6096491228070176 |
| Leaflet peduncle | 0.5877192982456141 |
| Leaf blade | 0.543859649122807 |
| Fruit peduncle | 0.5833333333333333 |
| Fruit pedicle | 0.7192982456140351 |
| Fruit flesh | 0.6228070175438596 |
| Seed | 0.5570175438596492 | Tissue
B
Genotype
### Chart
| Category | |
|---|---|
| 5-year-old root | 0.32894736842105265 |
| 12-year-old root | 0.2763157894736842 |
| 18-year-old root | 0.2894736842105263 |
| 25-year-old root | 0.3377192982456141 |Percentage of the PgZFP transcripts expressed at a development stage
Age
C
18-year-old root
12-year-old root
5
(4.6%)
4
(3.7%)
2
(1.8%)
4
(3.7%)
2
(1.8%)
14
(13%)
13
(12%)
2
(1.8%)
0
(0%)
46
(42.6%)
4
(3.7%)
3
(2.8%)
2
(1.8%)
5
(4.6%)
2
(1.8%)
25-year-old root
5-year-old root
Supplemental Figure S5. Percentage of transcripts in the PgZFP gene family expressed in a tissue (A), at a developmental stage (B), Venn diagram of numbers of the PgZFP transcripts expressed across developmental stages (C), and in a genotype (D).
