# Supplementary material for: Genome-wide analysis of the C2H2 zinc finger protein gene family and its response to salt stress in ginseng, Panax ginseng Meyer
Source: Sci Rep. 2022 Jun 17;12:10165. doi: 10.1038/s41598-022-14357-w (PMC9206012; doi:10.1038/s41598-022-14357-w)
Supplement: Supplementary file 6 — Supplementary Figure S6. [file 41598_2022_14357_MOESM6_ESM.pptx]

## Slide 1
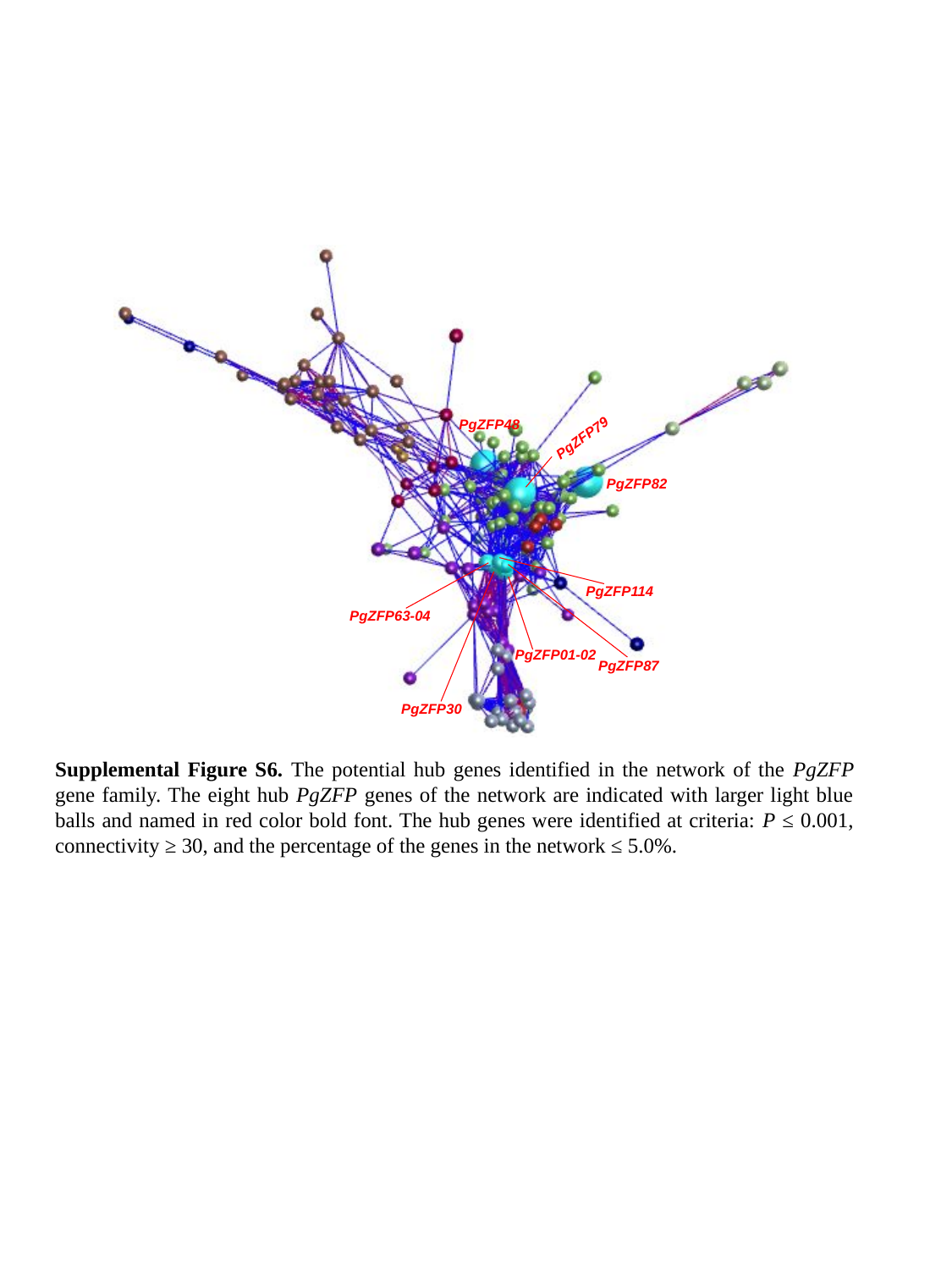

PgZFP48
PgZFP79
PgZFP82
PgZFP114
PgZFP63-04
PgZFP01-02
PgZFP87
PgZFP30
Supplemental Figure S6. The potential hub genes identified in the network of the PgZFP gene family. The eight hub PgZFP genes of the network are indicated with larger light blue balls and named in red color bold font. The hub genes were identified at criteria: P ≤ 0.001, connectivity ≥ 30, and the percentage of the genes in the network ≤ 5.0%.
